# Analysis of mutations leading to *para*-aminosalicylic acid resistance in *Mycobacterium tuberculosis*

**DOI:** 10.1038/s41598-019-48940-5

**Published:** 2019-09-20

**Authors:** Bharati Pandey, Sonam Grover, Jagdeep Kaur, Abhinav Grover

**Affiliations:** 1 Department of Biotechnology, Panjab University, 160014 Chandigarh, India; 2 JH-Institute of Molecular Medicine, Jamia Hamdard, Hamdard Nagar, 110062 New Delhi, India; 3 School of Biotechnology, Jawaharlal Nehru University, 110067 New Delhi, India

**Keywords:** Protein function predictions, Molecular modelling

## Abstract

Thymidylate synthase A (ThyA) is the key enzyme involved in the folate pathway in *Mycobacterium tuberculosis*. Mutation of key residues of ThyA enzyme which are involved in interaction with substrate 2′-deoxyuridine-5′-monophosphate (dUMP), cofactor 5,10-methylenetetrahydrofolate (MTHF), and catalytic site have caused *para*-aminosalicylic acid (PAS) resistance in TB patients. Focusing on R127L, L143P, C146R, L172P, A182P, and V261G mutations, including wild-type, we performed long molecular dynamics (MD) simulations in explicit solvent to investigate the molecular principles underlying PAS resistance due to missense mutations. We found that these mutations lead to (i) extensive changes in the dUMP and MTHF binding sites, (ii) weak interaction of ThyA enzyme with dUMP and MTHF by inducing conformational changes in the structure, (iii) loss of the hydrogen bond and other atomic interactions and (iv) enhanced movement of protein atoms indicated by principal component analysis (PCA). In this study, MD simulations framework has provided considerable insight into mutation induced conformational changes in the ThyA enzyme of *Mycobacterium*.

## Introduction

Antimicrobial resistance (AMR) threatens the effective treatment of tuberculosis (TB) caused by the bacteria *Mycobacterium tuberculosis* (Mtb) and has become a serious threat to global public health^1^. In 2017, there were reports of 5,58000 new TB cases with resistance to rifampicin (first line drug), of which 82% have developed multidrug-resistant tuberculosis (MDR-TB)^2^. AMR has been reported to be one of the top health threats globally, so there is an urgent need to proactively address the problem by identifying new drug targets and understanding the drug resistance mechanism^3,4^.

In 1902, Seidel and Bittner first synthesized and rediscovered the anti-TB activity of *para*-aminosalicylic acid (PAS)^5^. PAS was the first antituberculous drug used in combination therapy in the treatment of TB along with isoniazid and streptomycin and showed good outcome^6,7^. Recently, World Health Organization (WHO) has updated recommendations on the second line drugs used for the treatment of MDR-TB, in which PAS, Pyrazinamide, Ethambutol, Delamanid, Ethionamide, Streptomycin and Meropenem drugs have been group together in group C. Group C drugs should be used to complete the TB treatment when drugs from group A (Bedaquiline, Moxifloxacin, Linezolid, and Levofloxacin) and group B (Clofazimine, Cycloserine, and Terizidone) cannot be used^8^. PAS is a prodrug, having structural similarities with sulfonamides, which are structural analogues of para-aminobenzoic acid (PABA). Hence, PAS outperforms PABA as a substrate for dihydropteroate synthase (DHPS) (encoded by the *folP1/P2* gene), disrupting the *de novo* biosynthesis of folate^9^. Conversely, PAS was found to be a poor inhibitor of *M. tuberculosis* DHPS compared with dapsone, sulfamethoxazole, and sulfamethoxypyridazine^10^. PAS resistance was reported in strains of *M. tuberculosis* due to various missense mutations in the 7,8-dihydrofolate monoglutamate (H2Pte-Glu) binding pocket of the dihydrofolate synthase (DHFS) (encoded by the *folC* gene). DHFS is ultimate enzyme of the *de novo* folate synthesis pathway and is involved in the bioactivation of PAS^11,12^. Rengarajan *et al*.^13^ first reported that *Mycobacterium* develops PAS resistance due to mutations present in the *thymidylate synthase A* gene (*thyA*) using transposon mutagenesis^13^. Sequence analysis of *thyA* gene in 118 strains including laboratory references strains (H37Ra, H37Rv, *M. bovis*-NADL, *M. bovis*-Ravenel, BCG-Branch, BCG-Pasteur, *M. microti*, *M. africanum*), clinically diverse and clustered PAS^r^ (PAS-resistant) strains, and PAS^r^ spontaneous mutants, showed that thirty-seven percent of samples (PAS-resistant clinical isolates and spontaneous mutants) had mutations in the *thyA* gene and also pinpointed 24 distinct mutations including 4 in clinical isolates and 20 in spontaneous mutants respectively^14^. Furthermore, twenty novel PAS resistance mutations in *thyA* gene have been reported in the Mtb clinical isolates from Northern China^15^.

Mtb has two different types of thymidylate synthase (TS) enzymes, ThyA and ThyX. However, the Mtb genome with a deleted *thyA* gene conferred PAS resistance and showed defective *in vitro* growth but normal *in vivo* growth^16^. Therefore, it was reported that only ThyX expression was essential for normal growth of *M. tuberculosis*^17^. Moreover, humans have only ThyA enzyme and both, ThyA and ThyX TS enzymes from Mtb are structurally and evolutionarily unrelated which resulted in the discovery of the new inhibitors against ThyX^17–19^. The crystal structures of the ThyA enzyme in *Brucella melitensis* (PDBID: 3IX6), *M. tuberculosis* (PDBID: 4FOX and 3QJ7)^20^, *Enterococcus faecalis* (PDB IF: 6QYA), and *Escherichia coli* (PDB ID: 1AXW) have been solved at 2.2 Å, 2.3 Å, 1.76 Å, and 1.7 Å resolution, respectively. The active and dimerization domains of ThyA are formed by residues ranging from 21–209 and 16–205, respectively. In addition, C-terminal amino acid residues play role in the catalysis of reductive methylation^15^.

ThyA (EC 2.1.1.45) enzyme (encoded by the *thyA* gene) plays a significant role in thymine biosynthesis in folate pathway. ThyA enzyme catalyzes the conversion of 2′-deoxyuridine-5′-monophosphate (dUMP) to 2′ deoxythymidine-5′-monophosphate (dTMP) as follows:

1 $$5,\,10\,-\,{\rm{m}}{\rm{e}}{\rm{t}}{\rm{h}}{\rm{y}}{\rm{l}}{\rm{e}}{\rm{n}}{\rm{e}}{\rm{t}}{\rm{e}}{\rm{t}}{\rm{r}}{\rm{a}}{\rm{h}}{\rm{y}}{\rm{d}}{\rm{r}}{\rm{o}}{\rm{f}}{\rm{o}}{\rm{l}}{\rm{a}}{\rm{t}}{\rm{e}}\,+\,{\rm{d}}{\rm{U}}{\rm{M}}{\rm{P}}\,=\,{\rm{d}}{\rm{T}}{\rm{M}}{\rm{P}}\,+\,{\rm{d}}{\rm{i}}{\rm{h}}{\rm{y}}{\rm{d}}{\rm{r}}{\rm{o}}{\rm{f}}{\rm{o}}{\rm{l}}{\rm{a}}{\rm{t}}{\rm{e}}$$

ThyA also requires 5, 10-methylenetetrahydrofolate (MTHF) for dTMP synthesis both as a carbon donor and as a reductant in the methylation reaction^21^. Therefore, dTMP is important for DNA synthesis and loss of function mutations in the *thyA* gene causes PAS resistance in Mtb^13^.

Understanding of how point mutations induce structural changes in ThyA enzyme will provide opportunities for tackling PAS resistance. Therefore, we selected ThyA mutations (causing PAS resistance) correlated with minimum inhibitory concentration (MIC) values. Comparative analysis of the binding free energy, secondary structure elements, and free energy landscape of the wild-type and mutants was carried out using Molecular Dynamics (MD) simulations. The results of study showed that the wild-type ThyA enzyme formed strong interaction with dUMP (substrate) and MTHF (cofactor) mainly by hydrogen and hydrophobic interactions while, mutations apparently disturb the ThyA protein structure, making it dysfunctional.

## Results

The emergence of mutation causing antibiotic resistance in the *Mycobacterium* necessitates inspecting the molecular mechanism underlying resistance. Extensive MD simulations were carried out to differentiate systematic changes in the wild-type and mutant ThyA enzyme structures and provide substantial insight into the mechanism underlying PAS resistance.

### Selection of mutants

The mutants were selected based on MIC value ≥128 µg/ml^14^. Mutated residues were reported to be involved in interaction with substrate dUMP (R127L), catalytic site (C146R), MTHF cofactor (L143P, L172P and V261G), and completely buried in the hydrophobic core (A182P) of the wild-type ThyA enzyme^14^, as mentioned in Table 1. The selected mutations were also reported to destabilize the structure based on their positive folding free energy (∆∆G (kcal/mol)^14^ (Table 1). Therefore, structural transitions due to point substitution in ThyA enzyme was discussed specifically using atomistic studies.

**Table 1:** Energetic parameters computed for the ThyA wild-type and mutants.

Systems	Structural features	MIC (μg/ml)	∆∆G (kcal/mol)	Born self-energy (kcal)	Coulomb energy (kcal)	Electrostatic solvation energy (kcal/mol)	Total energy (kcal/mol)
Wild-type	—	—	—	−4904.69	−33072.95	−1026.18	−33486.94
R127L	In interaction with substrate dUMP	>128	+0.18	−4041.04	−33263.96	−646.37	−33317.70
L143P	In interaction with THF cofactor	>128	+2.45	−4013.56	−33249.83	−582.75	−33245.12
C146R	Catalytic residue	>128	+1.56	−4060.56	−33371.97	−651.38	−33452.05
L172P	In interaction with THF cofactor	>128	+1.28	−4951.73	−32861.77	−1157.29	−33392.49
A182P	Completely buried into the hydrophobic core of the enzyme	>128	+3.94	−4030.89	−33348.92	−656.03	−33413.51
V261G	In interaction with THF cofactor	>128	+0.52	−4995.59	−32856.69	−1180.65	−33407.43

### Molecular dynamics (MD) simulation analysis

**Figure 1:**
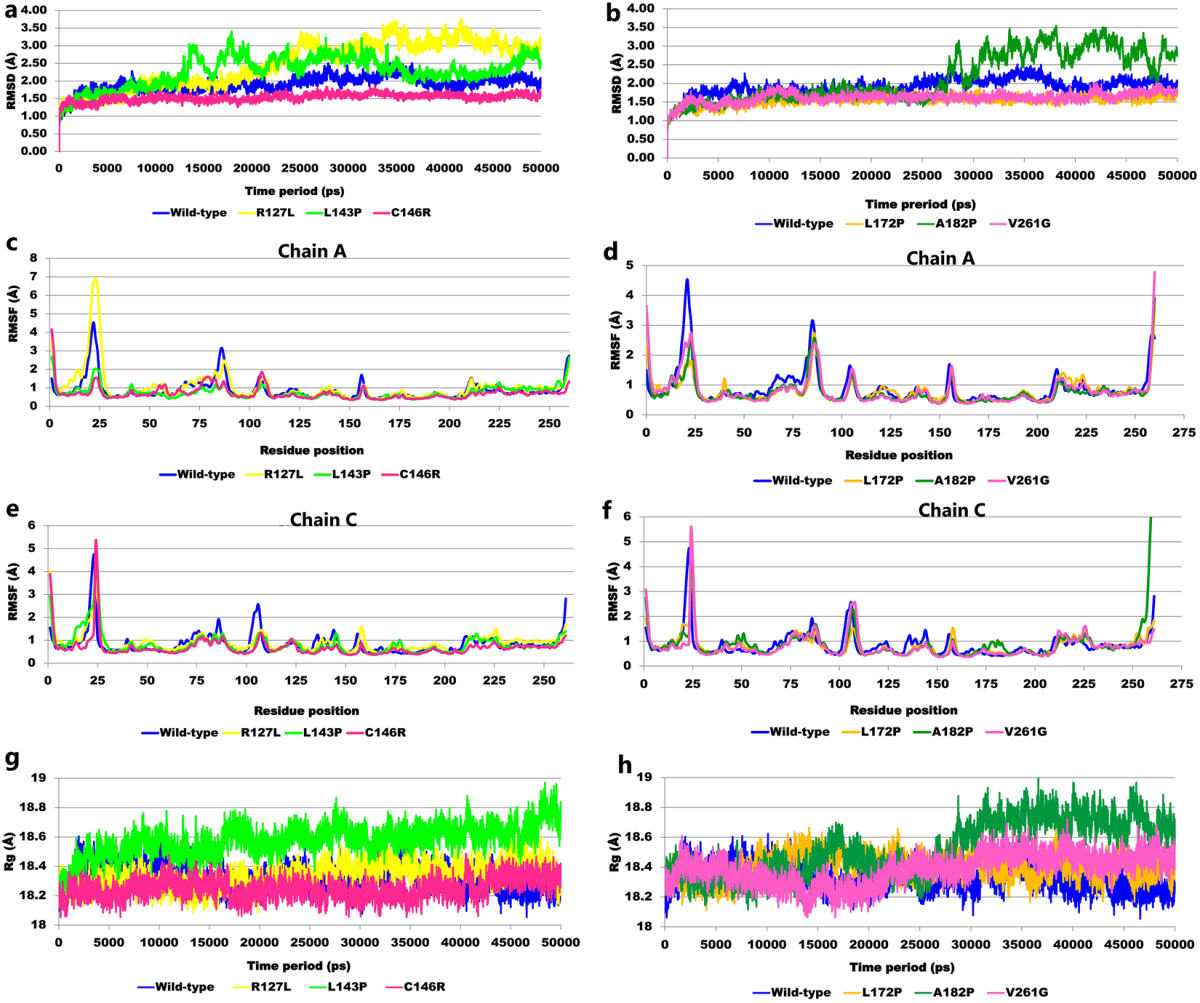
MD trajectory analysis of the ThyA wild-type and mutants: (**a**,**b**) root mean square deviation analysis (**c–f**) per-residue root mean square fluctuation analysis for both chain A and C (**g**,**h**) radius of gyration.

All systems were simulated for 50 ns that helped in achieving much better conformational coverage for the wild-type and mutants. The homo-2-mer structure of the ThyA enzyme has 4 polypeptide chains, A and B chains with 260 amino acid residues and C and D with 261 residues. We selected dimer A-C for the present study, and chain A of the dimer showed interaction with the dUMP substrate and MTHF cofactor, respectively. MD simulation trajectories representing all-atomic coordinates as a function of time were analyzed for all seven systems: wild-type, R127L, L143P, C146R, L172P, A182P, and V261G. Furthermore, the average root-mean-square deviations (RMSDs) for wild-type, R127L, L143P, C146R, L172P, A182P, and V261G were found to be 1.91, 2.49, 2.27, 1.53, 1.55, 2.16, and 1.61 Å, respectively (Table 2; Fig. 1(a,b)). The RMSD values for wild-type and mutants were <3 Å, which is acceptable, implying that the systems were converged^22^. However, higher RMSD values were observed for R127L, L143P, and A182P complexes than the wild-type, which represented large conformational change during the simulation run. R127L and L143P were unstable at the beginning and attained stability between 35 and 45 ns. However, the A182P complex remained stable up to 25 ns, and then become highly unstable until the end of the simulations.

**Table 2:** Averaged properties calculated from trajectories of ThyA wild-type and mutants.

Systems	Avg. RMSD(Å)	Avg. RMSF(Å)		Avg. Rg (Å)	Avg. Hbond-dUMP	Avg. Hbond-MTHF
		Chain A	Chain C			
Wild-type	1.91	0.88	0.83	18.33	5	1
R127L	2.49	1.07	0.90	18.34	4	—
L143P	2.27	0.75	0.81	18.58	5	—
C146R	1.53	0.71	0.69	18.25	4	6
L172P	1.55	0.81	0.81	18.40	5	1
A182P	2.16	0.78	0.92	18.52	4	2
V261G	1.61	0.81	0.80	18.38	5	1

Avg; Average.

The root mean square fluctuation (RMSF) plot explains flexibility of each amino acid residue providing insight into flexible and rigid regions of the protein. The RMSF plot of the wild-type and mutants for chain A and C are shown in Fig. 1(c–f). The average RMSF values for the wild-type for chain A and C were 0.88 Å and 1.04 Å respectively. Furthermore, the average RMSF for mutants ranged from 0.81–1.07 Å and 0.80–0.92 Å for chain A and C, respectively (Table 2). The highest fluctuation peaks (>3 Å) for wild-type and R127L were located from 15–25 residue position in chain A. In chain C, higher RMSF values (>3 Å) were shown by wild-type, R127L, C146R, and V261G complexes, in residues ranging from 15–25 respectively. From the RMSF plot and values, we observed that mutation positions in the respective mutants showed lower flexibility (relative to wild-type), which may cause improper binding of the substrate and cofactor, which is required for proper enzymatic activity (Table S1).

The radius of gyration (Rg) was computed for all systems which reflected a change in global protein compactness due to point mutation^23^. The Rg was computed using following equation

2 $$Rg=\sqrt{\frac{1}{{N}_{i}}{{\sum }_{i}({r}_{i}-{r}_{cm})}^{2}}$$

where *r* is the position of atom *i*, and r_*i*_ − r_cm_ is the distance between atom i and the center of mass of the molecule.

The average Rg values for the wild-type, R127L, L143P, C146R, L172P, A182P, and V261G were 18.33, 18.34, 18.58, 18.25, 18.40, 18.52, and 18.38 Å, respectively. All mutants showed greater Rg values than the wild-type, with the exception of C146R, suggesting tight packing of the secondary structure (α, β, α + β, α/β) into the three-dimensional structure of wild-type ThyA enzyme (Table 2; Fig. 1(g,h)).

**Figure 2:**
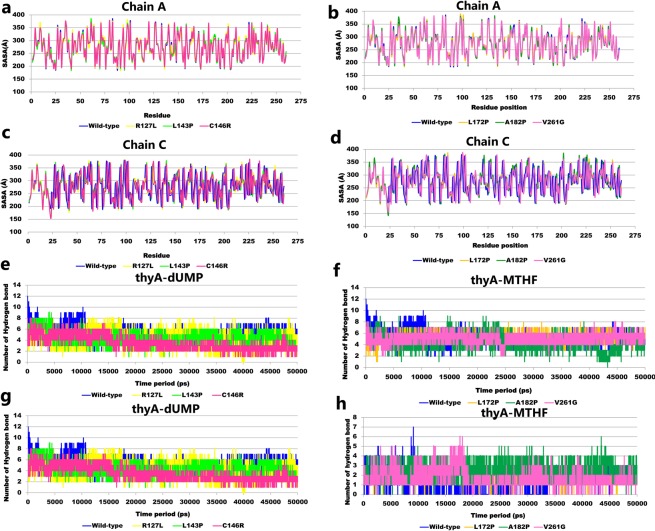
MD trajectories analysis of the ThyA wild-type and mutants: (**a**–**d**) solvent accessible surface area analysis for both chain A and C (**e**,**f**) number of inter molecular hydrogen bonds interaction between ThyA and dUMP (**g**,**h**) number of inter molecular hydrogen bonds interaction between ThyA and MTHF.

Determination of the residue-wise solvent-accessible surface area (SASA) reflected the change in the surface organization of the mutants compared with the wild-type. In R127L, leucine substituted natively placed arginine which may decrease protein stability due to leucine’s aversion to water and a reduced SASA value from 372.07 (wild-type) to 348.26 (R127L) was observed. Substitution of the non-polar group (leucine, alanine and valine) by proline (hydrophobic side chain) and glycine (without side chain) led to slight decreases in the SASA values in L143P, L172P, A182P, and V261G mutants compared with the wild-type. Furthermore, a change from the polar uncharged group to the polar charged group increased the SASA value at the 146 position from 259.44 (wild-type) to 369.41 (C146R) (Fig. 2(a–d)).

To estimate the contribution of hydrogen bonds (H-bonds) to ThyA enzyme complex stability, the total number of H-bonds was computed during MD simulations. A total of five H-bonds were observed between dUMP and ThyA for wild-type, L143P, L172P, and V261G, respectively, as compared with four for the R127L, C146R, and A182P complexes. However, a single H-bond was observed between MTHF and ThyA in wild-type, L172P, and V261G, respectively. No H-bonds were detected in MTHF bound R127L and L143P complexes, while C146R-MTHF complex showed the maximum number of H-bonds during the 50 ns simulation time period (Table 2; Fig. 2(e–h)).

### Binding pocket analysis

Binding pocket analysis was carried out to interpret the variation in the volume and area of the protein with respect to the simulation time series. Initially, the structure conformation (at 0 ns) of the wild-type, R127L, L143P, C146R, L172P, A182P, and V261G mutants remained same. The volume of mutants R127L, L143P, C146R, L172P, and V261G (representative structures) was found to increase when compared with the wild-type, except the A182P mutant (Table 3).

**Table 3:** Analysis of enzyme binding cavity for ThyA wild-type and mutants.

Time	0 ns	10 ns	20 ns	30 ns	40 ns	50 ns	Representative Structure
**Wild-type**							
Volume (Å^3^)	804.6	1189.2	1108.2	578.1	1163.5	1597.0	710.0
Area (Å^2^)	639.5	1071.8	917.9	520.1	825.6	931.2	758.6
**R127L**							
Volume (Å^3^)	804.6	906.7	866.1	970.4	1217.8	924.6	901.2
Area (Å^2^)	639.5	792.7	784.1	927.8	873.5	748.1	686.7
**L143P**							
Volume (Å^3^)	804.6	866.1	970.4	723.6	772.0	415.1	1012.0
Area (Å^2^)	639.5	784.1	927.8	711.6	718.6	425.8	780.7
**C146R**							
Volume (Å^3^)	804.6	944.7	1004.1	1261.7	1164.2	1123.0	947.6
Area (Å^2^)	639.5	639.5	823.9	929.5	807.2	808.0	865.5
**L172P**							
Volume (Å^3^)	804.6	864.8	925.7	561.3	722.8	722.8	1190.3
Area (Å^2^)	639.5	611.5	650.7	521.3	586.0	586.0	855.6
**A182P**							
Volume (Å^3^)	804.6	599.2	899.1	215.5	368.6	366.3	562.5
Area (Å^2^)	639.5	468.5	645.5	385.3	412.0	440.2	358.6
**V261G**							
Volume (Å^3^)	804.6	1748.8	1557.8	1458.3	2140.4	1578.4	1466.4
Area (Å^2^)	639.5	1224.5	939.3	951.8	1279.4	990.6	1005.4

The drastic increase in the binding pockets could have led to an increase formation of H-bond between dUMP and mutants (L143P, L172P, and V261G) and a reduction for MTHF, which was in agreement with the H-bond analysis with respect to time. Interestingly, the representative structure of the A182P mutant showed an extreme decrease in its binding volume and area compared with its initial structure and wild-type representative structure and, accordingly, showed reduced H-bond occupancy for dUMP and an increase for the MTHF cofactor (Table 3). It was observed that even a single mutation could lead to too many differences in the binding affinity of enzyme substrate and cofactor.

### Secondary structure analysis

The secondary structure analysis (helix, β-strand, and coil) provided information about the conformation of each amino acid in the representative structure of ThyA wild-type and mutants. The secondary structure elements (SSEs) are of two types: rigid (α-helices and β-sheet) and flexible (coil and turns). The wild-type was dominated by turns (28.85%) in chain A and α-helices (29.73%) in chain C (Table 4). The C146R, A182P, and V261G mutants showed increases in coils in chain A and in A182P and V261G in chain C, respectively, as compared with the wild-type. Chain A showed increase in α-helices in R127L, L143P, C146R, L172P, A182P, and V261G mutants, whereas minimal deviation was observed in α-helix SSEs in chain C for all mutants with respect to the wild-type. In case of β-sheet, all mutants showed increases in β-sheet with the exception of L172P in chain A, and R127L, C146R, and A182P showed increases in β-sheet in chain C compared with the wild-type. Both L143P and  L172P mutants showed an increase in turn structure in chain A while all the mutants showed increases in turn in chain C relative to the wild-type. Furthermore, decreases in the percentages of 3_10_-helix SSEs were observed in chain A and C in all mutants compared with the wild-type (Table 4).

**Table 4:** Comparative secondary structure elements analysis of the ThyA wild-type and mutants.

Systems	Coil (C)%		α-Helix (H)%		β-Sheet (E)%		Turn (T)%		3_10_-Helix (G)%		Isolated β-bridge (B)%	
	Ch_A	Ch_C	Ch_A	Ch_C	Ch_A	Ch_C	Ch_A	Ch_C	Ch_A	Ch_C	Ch_A	Ch_C
Wild-type	17.31	18.92	25.38	29.73	23.85	23.94	28.85	21.24	4.62	4.63	—	1.54
R127L	15.77	16.99	27.69	29.34	26.15	25.10	25.00	25.48	4.62	2.32	0.77	0.77
L143P	16.92	18.77	27.69	29.11	24.23	23.37	29.61	24.52	1.15	3.46	0.77	0.77
C146R	18.08	18.15	30.00	29.73	26.15	26.25	23.85	23.94	1.15	1.16	0.77	0.77
L172P	17.31	17.76	25.77	29.73	23.85	22.78	29.23	28.96	3.46	—	0.38	0.77
A182P	17.69	19.62	28.85	30.00	25.00	24.23	25.00	23.46	3.46	2.31	—	0.38
V261G	19.23	21.07	28.08	29.12	24.62	18.77	26.92	26.44	1.15	2.30	0.38	2.30

Ch; Chain.

In the wild-type, residues at positions 127, 143, 146, 172, 182, and 261 located in α-helix in both chain A and C. The R127L, L143P, C146R, and L172P mutants showed transformation into turn compared with the wild-type in both chain A and chain C. The A182P mutant retained its SSE, whereas V261G mutants showed transformation from turn to coil in chain C. It was observed from the results that mutated residue showed a change from a rigid to a flexible secondary structure (Table S2).

### Residue network analysis

A residue network graph of the ThyA enzyme bound with dUMP and MTHF was visualized for analyzing and depicting impact of mutation on interacting residues. Residue 127 formed van der Waals interactions in R127L, C146R, L172P, A182P, and V261G complexes, respectively, and single H-bonds in A182P and V261G complexes (Table 5). Three van der Waals interactions were formed by residue 143 in the wild-type, A182P, and V261G; four in C146R and L172P complexes; and two in R127L and L143P, respectively. Van der Waals Interaction 143-80 was found to be lost in L143P and 143-93 interaction in A182P and V261G complexes. In the wild-type and mutants of ThyA, a single H-bond was formed between 146 and 172 residues. Residue 182 formed a single H-bond in the wild-type and mutant forms of ThyA (except A182P), while two in C146R. In addition, all the complexes were extensively stabilized by the van der Waals forces formed by residue 182. Residue 261 stabilized only the L172P complex through the formation of a single van der Waals interaction (261–211) (Table 5). In addition, complete list of ThyA residues interacting with the dUMP and MTHF in the interaction network of wild-type and mutants are listed in Table S3.

**Table 5:** Interaction of key residues in ThyA wild-type and mutant complexes.

Interactions	Wild-type	R127L	L143P	C146R	L172P	A182P	V261G
127-118	—	VDW	—	VDW	VDW	VDW	VDW
127-122	—	—	—	—	—	H. bond	H. bond
143-83	VDW	VDW	VDW	VDW	VDW	VDW	VDW
143-84	—	—	—	—	—	—	VDW
143-80	VDW	VDW	—	VDW	VDW	VDW	VDW
143-90	—	—	—	VDW	VDW	VDW	—
143-93	VDW	—	VDW	VDW	VDW	—	—
146-94	H. bond	H. bond	H. bond	H. bond	H. bond	H. bond	H. bond
146-143				VDW	—	—	—
172-169	H. bond	H. bond	H. bond	H. bond, VDW	H. bond, VDW	H. bond	H. bond
172-48	—	—	—	—	—	VDW	—
182-178	H. bond	H. bond	H. bond	H. bond	H. bond	—	H. bond
182-179	—	—	—	H. bond	—	VDW	—
182-42	VDW	VDW	VDW	VDW	VDW	VDW	VDW
182-43	—	VDW	VDW	VDW	VDW	VDW	VDW
182-38	—	VDW	—	VDW	VDW	—	—
261-211					VDW		

*“VDW”: van der Waals, “—”: not present.

### Electrostatic potential and binding affinity analysis

**Figure 3:**
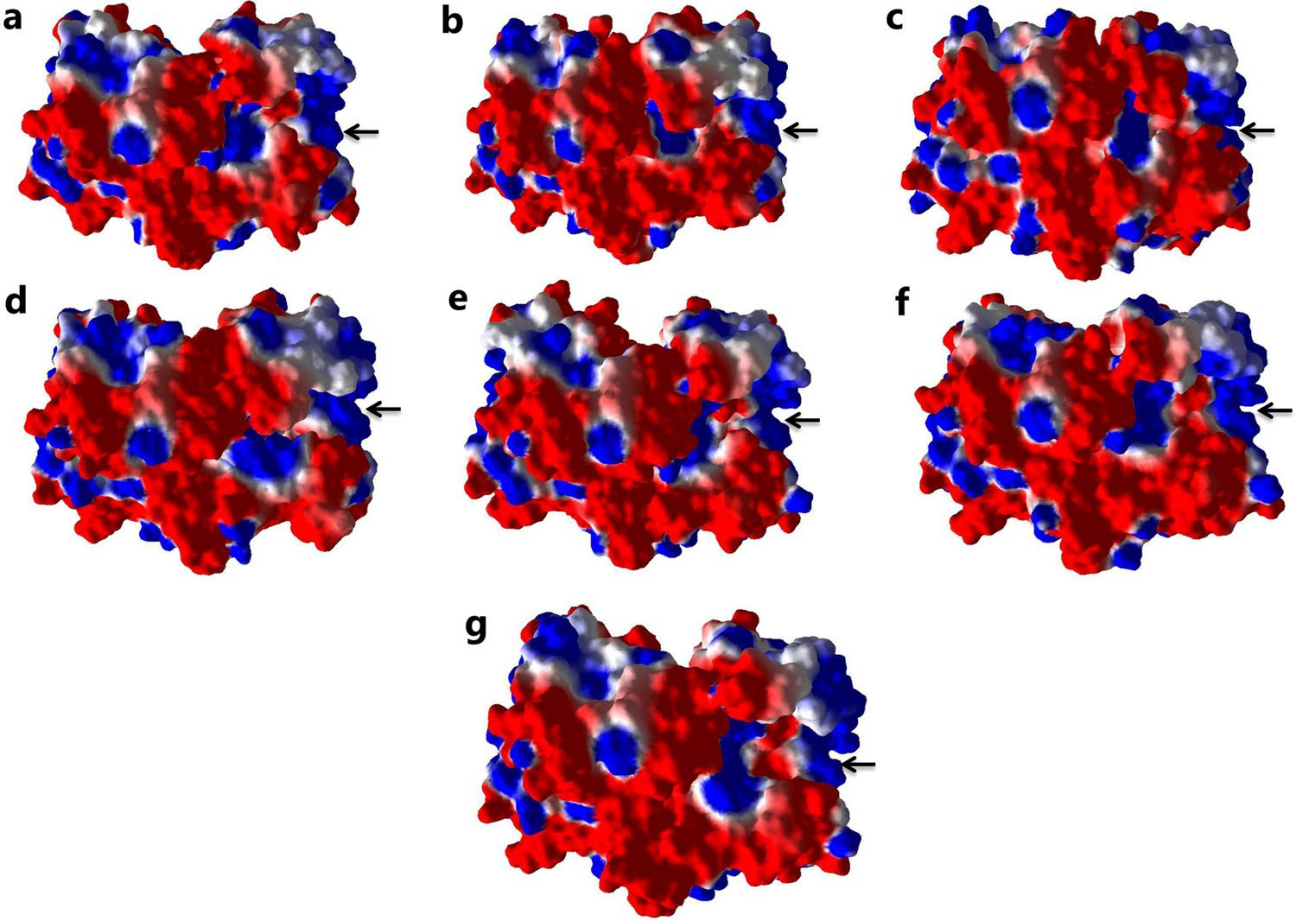
Electrostatic surface potential of ThyA (**a**) wild-type, (**b**) R127L, (**c**) L143P, (**d**) C146R, (**e**) L172P, (**f**) A182P and (**g**) V261G mutants. Red symbolizes negative charge; blue positive and white is neutral. Arrow indicates dUMP and MTHF binding site in chain A of ThyA enzyme.

The estimation of electrostatic properties based on Generalized Born radii provided insight into the effect of point mutation on the ThyA enzyme structure and stability. Figure 3 shows the electrostatic potential surface for the wild-type and mutants. Furthermore, changes in the enzyme stability upon mutation were computed in terms of total energy, where a positive value signifies an unstable structure and a negative value signifies a stable structure. The wild-type exhibited total energy of −33486.94 kcal/mol, whereas the R127L, L143P, C146R, L172P, A182P, and V261G mutants showed total energy ranging from −33245.12 to −33452.05 kcal/mol, respectively, higher than that of the wild-type. The results were in accordance with the previous studies that have reported positive folding free energy (∆∆G (kcal/mol)) ranging from +0.18 to +3.94 for R127L, L143P, C146R, L172P, A182P, and V261G mutations (Fig. 3; Table 1)^14^.

**Figure 4:**
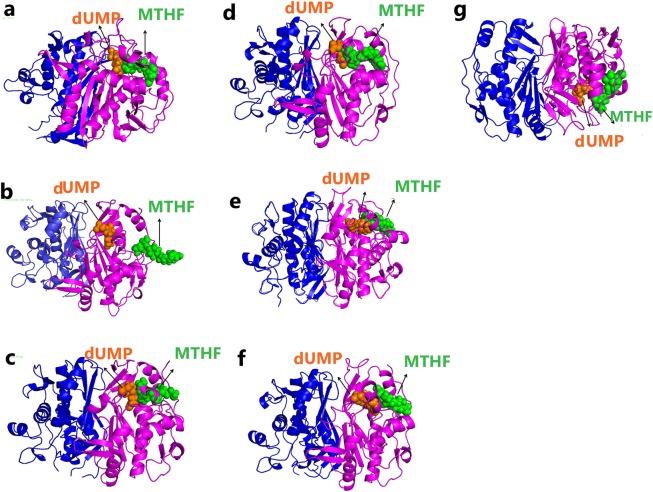
Ribbon representation of the ThyA enzyme structure bounded with substrate dUMP and cofactor MTHF for (**a**) wild-type, (**b**) R127L, (**c**) L143P, (**d**) C146R, (**e**) L172P, (**f**) A182P and (**g**) V261G mutants.

In addition, binding energies of the ThyA enzyme-substrate and cofactor complexes were calculated before and after MD simulations through the Molecular Mechanics/Generalized Born Surface Area (MM/GBSA) approach. In the pre-MD ThyA-dUMP complexes, the highest binding affinity was displayed in the wild-type complex (−15.37 kcal/mol), followed by the A182P mutant complex (−10.47 kcal/mol), while the lowest binding affinity was observed in the R127L and V261G mutant complexes (Table 6). However, in the post-MD ThyA-dUMP complexes, dUMP showed the strongest binding affinity for the wild-type (−15.11 kcal/mol), followed by the L143P (−8.06 kcal/mol) and C146R (−7.53 kcal/mol) complexes. Conversely, the L172P and V261G mutant complexes showed positive binding free energy, suggesting weak interaction between dUMP and the ThyA enzyme (Fig. 4; Table 6).

**Table 6:** Binding energy analysis in the pre- and post-MD simulation complexes in the ThyA wild-type and mutants.

MM-GBSA Binding energy (kcal/mol)	Wild-type		R127L		L143P		C146R		L172P		A182P		V261G	
	dUMP	MTHF	dUMP	MTHF	dUMP	MTHF	dUMP	MTHF	dUMP	MTHF	dUMP	MTHF	dUMP	MTHF
Pre-MD	−15.37	−8.70	−4.76	−7.99	−7.33	−8.09	−5.05	−5.51	−5.10	−11.39	−10.47	−8.92	−2.54	−13.43
Post-MD	−15.11	−20.22	−4.95	−6.05	−8.06	−5.00	−7.53	−11.35	+21.34	−1.33	−6.68	−15.24	+4.67	−7.22

In pre-MD complexes, MTHF showed maximum binding affinity for ThyA in the V261G (−13.43 kcal/mol) and L172P (−11.39 kcal/mol) mutants, followed by the A182P (−8.92 kcal/mol) and wild-type (−8.70 kcal/mol) complexes. The R127L and V261G complexes showed the lowest binding free energy. In the post-MD complexes, wild-type ThyA enzyme-MTHF complex showed highest binding energy (−20.22 kcal/mol), followed by the A182P (−15.24 kcal/mol) and C146R (−11.35 kcal/mol) complexes. L172P and L143P complexes showed lowest binding free energy with −1.33 and −5.00 kcal/mol, respectively. The binding energy results showed that the point mutation at the dUMP binding site (127) and the MTHF binding site (143 and 172) had decreased the substrate and cofactor affinities for the ThyA enzyme, leading to weak binding of dUMP and MTHF (Table 6).

### Interaction analysis using MD trajectories for ThyA-dUMP interaction

To gain insight into the interactions between enzymes and their substrate and cofactors, the complexes were clustered using the MD trajectories, and representative structures were selected from wild-type and mutant complexes. In the wild-type, dUMP interacted via H-bonds with Tyr94, Asp166, Asp167, Asp169, Asn177, His207 (chain A), Arg126, and Arg127 (chain C) with H-bond occupancies of 34%, 92%, 31%, 48%, 31%, 60%, 90%, and 78%. The residue Arg166 belonged to the β-sheet and Asn177 and His207 to α-helix. Asp167, Asp169, Arg126, and Arg127 were located in the loop region. The six residues — Trp80, Leu143, Cys146, His147, Ala168, and Gly173 — also stabilized the ThyA-dUMP complex by forming a hydrophobic network (Fig. S1; Table 7).

**Table 7:** Residues participating in the interaction in the ThyA enzyme-dUMP complex in wild-type and mutants.

Binding site	No. of hydrogen bond	Participating residues with their bond length (Å)			Participating residues in hydrophobic bonding (Å)
Wild-type	10	**Acceptor**	**Donor**	**Occupancy (%)**	Trp80, Leu143, Cys146, His147, Ala168, Gly173, MTHF
		Asp167-OG	dUMP-O3	48%	
		Asp166-NH1	dUMP-OP2	92%	
		Asp166-NH2	dUMP-OP3	87%	
		Tyr94-OH	dUMP-O4	34%	
		Asn177-OD1	dUMP-N3	35%	
		Asn177-HD22	dUMP-O4	60%	
		His207-NE2	dUMP-O3	31%	
		Arg126-NH1	dUMP-OP1	90%	
		Arg127-NH1	dUMP-OP2	78%	
		Asp169-H	dUMP-O2	31%	
R127L	6	Arg126-NH1	dUMP-OP2	43%	Cys146, His147, Gln165, Ala168, Asn177, Tyr209
		Arg166-NH1	dUMP-OP3	91%	
		Gly173-O	dUMP-N3	40%	
		Asp167-OG	dUMP-OP1	44%	
		Asp169-H	dUMP-O2	47%	
		His207-NE2	dUMP-O3	45%	
L143P	5	Tyr209-OH	dUMP-O3	35%	His147, Ala168, Val174, MTHF
		Gln165-HE22	dUMP-O4	63%	
		Gly173-O	dUMP-NH3	49%	
		Trp83-HE1	dUMP-OP2	33%	
		Ile79-O	dUMP-HO3	41%	
C146R	6	Arg146-NH1	dUMP-OP2	62%	Gln165, Gly173, MTHF
		Arg166-NH2	dUMP-OP2	33%	
		Arg166-NH2	dUMP-OP2	100%	
		Ser167-OG	dUMP-OP1	30%	
		His207-NE2	dUMP-O3	34%	
		Tyr209-OH	dUMP-O3	32%	
L172P	7	Arg166-NH2	dUMP-OP2	100%	Ile79, Trp80, Trp83, Cys146, Arg166, Asp169, Asn177
		Arg166-NH2	dUMP-OP3	92%	
		Ser167-OG	dUMP-OP2	54%	
		Asp169-N	dUMP-O3	67%	
		Asn177-ND2	dUMP-O2	96%	
		Glu58-OE1	dUMP-NH3	44%	
		Trp83-NE1	dUMP-OP1	67%	
A182P	4	Asn177-ND2	dUMP-OD1	71%	Ile79, Trp80, Trp83, Cys146, Ser167, Gly173, Phe176
		Asn177-ND2	dUMP-O4	89%	
		Asp169-N	dUMP-O3	43%	
		Arg166-NH2	dUMP-OP3	80%	
V261G	6	Arg166-NH2	dUMP-OP2	96%	Ile79, Trp80, Trp83, Cys146, His147
		Arg166-NH2	dUMP-OP2	97%	
		Ser167-OG	dUMP-OP3	36%	
		Trp83-NE1	dUMP-OP3	87%	
		Asn177-ND2	dUMP-O3	88%	
		Glu58-OE1	dUMP-N3	97%	

In R127L, dUMP formed six H-bonds with Arg166 (91%), Asp167 (44%), Asp169 (47%), Gly173 (40%), and His207 (45%) residues from chain A and Arg126 (43%) residue from chain C of ThyA enzyme. Arg166, Asp167, Asp169, and His207 lie in the β-sheet and Gly173 and Arg126 in the loop region. The R127L-dUMP complex was stabilized with six hydrophobic interactions by Cys146, His147, Gln165, Ala168, Asn177, and Tyr209 amino acid residues. The L143P complex was stabilized by five H-bonds with Tyr209 (35%), Gln165 (63%), Gly173 (49%), Trp83 (33%), and Ile79 (41%) and three residues, namely His147, Ala168, and Val174, forming hydrophobic interactions. Moreover, the C146R complex was stabilized by H-bonds formed by Arg146 (62%), Arg166 (33% and 100%), Ser167 (30%), His207 (34%), and Tyr209 (32%) residues, and two residues involved in hydrophobic interactions were Gln165 and Gly173. The L172P complex was stabilized by H-bond formed by Arg166 (100% and 92%), Ser167 (54%), Asp169 (67%), Asn177 (96%), Glu58 (44%), and Trp83 (67%) residues, and hydrophobic interactions were formed by Ile79, Trp80, Trp83, Cys146, Arg166, Asp169, and Asn177 residues. In the A182P complex, four H-bonds were formed by Asn177 (71% and 89%), Asp169 (43%), Arg166 (80%) residues. The V261G complex was stabilized by Arg166 (96% and 97%), Ser167 (36%), Trp83 (87%), Asn177 (88%), Glu58 (97%) residues and five hydrophobic interactions (Table 7).

Consequently, at the point of mutation, the intensity of hydrophobic interactions remained unchanged while the H-bond number reduced drastically, indicating conformational changes in the mutant structures.

### Interaction analysis using MD trajectories for ThyA-MTHF interaction

In the wild-type, MTHF formed three H-bonds with Ile79, Lys49, and Leu172 with occupancies of 33%, 33%, and 31%, along with five hydrophobic interactions. R127L-MTHF formed two H-bonds with Lys49 (48%) and His51 (77%), and Val50, Phe176, and Ala256 residues formed hydrophobic interactions (Table 8). The L143P complex was stabilized by H-bonds formed by Ile79 and Lys48 (60%) and large numbers of residues, including Lys48, Trp80, Trp83, Phe171, Leu172, Gly173, Phe176, Ala256, Ile257, Lys258, stabilizing the complex by hydrophobic interactions. The interaction between C146R and MTHF was stabilized by H-bonds formed by Lys48 (31%), Gly173 (62%), His51 (84%), Lys49 (56%), Gln165 (36%), Arg146 (54%), Glu58 (50%), Leu172 (43%), and Ile257 (32%), and hydrophobic interactions were contributed by Val50, Ile79, Trp80, Trp83, Phe171, Pro175, and Phe176 residues. Likewise, the L172P mutant complex was stabilized by four residues — Lys48 (51%), Lys49 (99%), Ile79 (40%), and  Ile257 (46%) — and eight residues contributed hydrophobic interactions. Amino acid residues involved in the hydrogen and hydrophobic interactions with MTHF in A182P and V261G mutant complexes are listed in Table 8. The interaction analysis revealed that mutation at the 127 and 143 positions enforced large structural changes, leading to shifts in the MTHF away from its binding pocket.

**Table 8:** Residues participating in the interaction in the ThyA enzyme-MTHF complex in wild-type and mutants.

Binding site	No. of hydrogen bond	Participating residues with their bond length (Å)			Participating residues in hydrophobic bonding
Wild-type	3	**Acceptor**	**Donor**		Val50,Trp80, Trp83, Phe176, Ala259
		Ile79-N	MTHF-O1	33%	
		Lys49-NZ	MTHF-OE2	33%	
		Leu172-O	MTHF-O4	31%	
R127L	2	Lys49-NZ	MTHF-O	48%	Val50, Phe176, Ala256
		His51-N	MTHF-OE1	77%	
L143P	2	Ile79-O	MTHF-N10	47%	Lys48, Trp80, Trp83, Phe171, Leu172, Gly173, Phe176, Ala256, Ile257, Lys258
		Lys48-NZ	MTHF-O1	60%	
C146R	9	Lys48-NZ	MTHF-O1	31%	Val50, Ile79, Trp80, Trp83, Phe171, Pro175, Phe176
		Gly173-NZ	MTHF-O2	62%	
		His51-N	MTHF-OE2	84%	
		Lys49-NZ	MTHF-OE2	56%	
		Gln165-OE1	MTHF-NH3	36%	
		Arg146-HE	MTHF-N1	54%	
		Glu58-OE1	MTHF-NH8	50%	
		Leu172-O	MTHF-O4	43%	
		Ile257-O	MTHF-OE1	32%	
L172P	4	Lys48-NZ	MTHF-OE1	51%	Val50, His51, Ser54, Thr78, Glu82, Trp83, Phe176, Ala256
		Lys49-O	MTHF-OE1	99%	
		Ile79-O	MTHF-O4	40%	
		Ile257-H	MTHF-OE1	46%	
A182P	4	Lys48-NZ	MTHF-O1	49%	Val50, Ser54, Thr78, Ala256
		Lys49-O	MTHF-OE1	98%	
		His51-H	MTHF-O	50%	
		Leu172-O	MTHF-O4	51%	
V261G	3	Lys48-NZ	MTHF-OE1	40%	Lys48, His51, Thr78, Glu82, Phe176, Ala256
		Lys49-O	MTHF-OE1	97%	
		Ile257-O	MTHF-OE1	33%	

### Principle component and Free energy analysis

**Figure 5:**
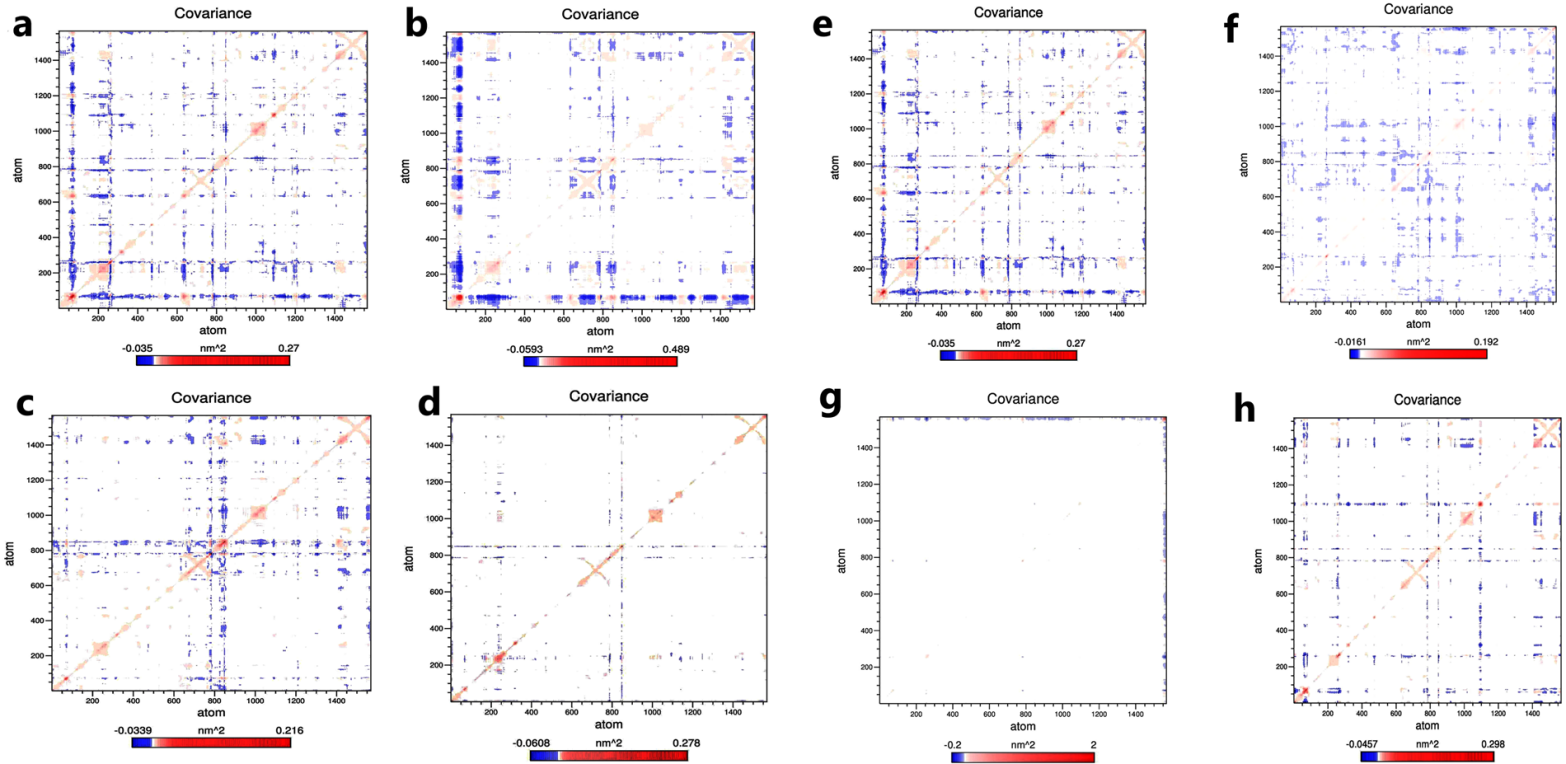
Covariance matrix plot for the ThyA bound with substrate dUMP and cofactor MTHF (**a** and **e**) wild-type, (**b**) R127L, (**c**) L143P, (**d**) C146R, (**f**) L172P, (**g**) A182P and (**h**) V261G mutants. Red and blue to positive and negative correlated residues.

**Figure 6:**
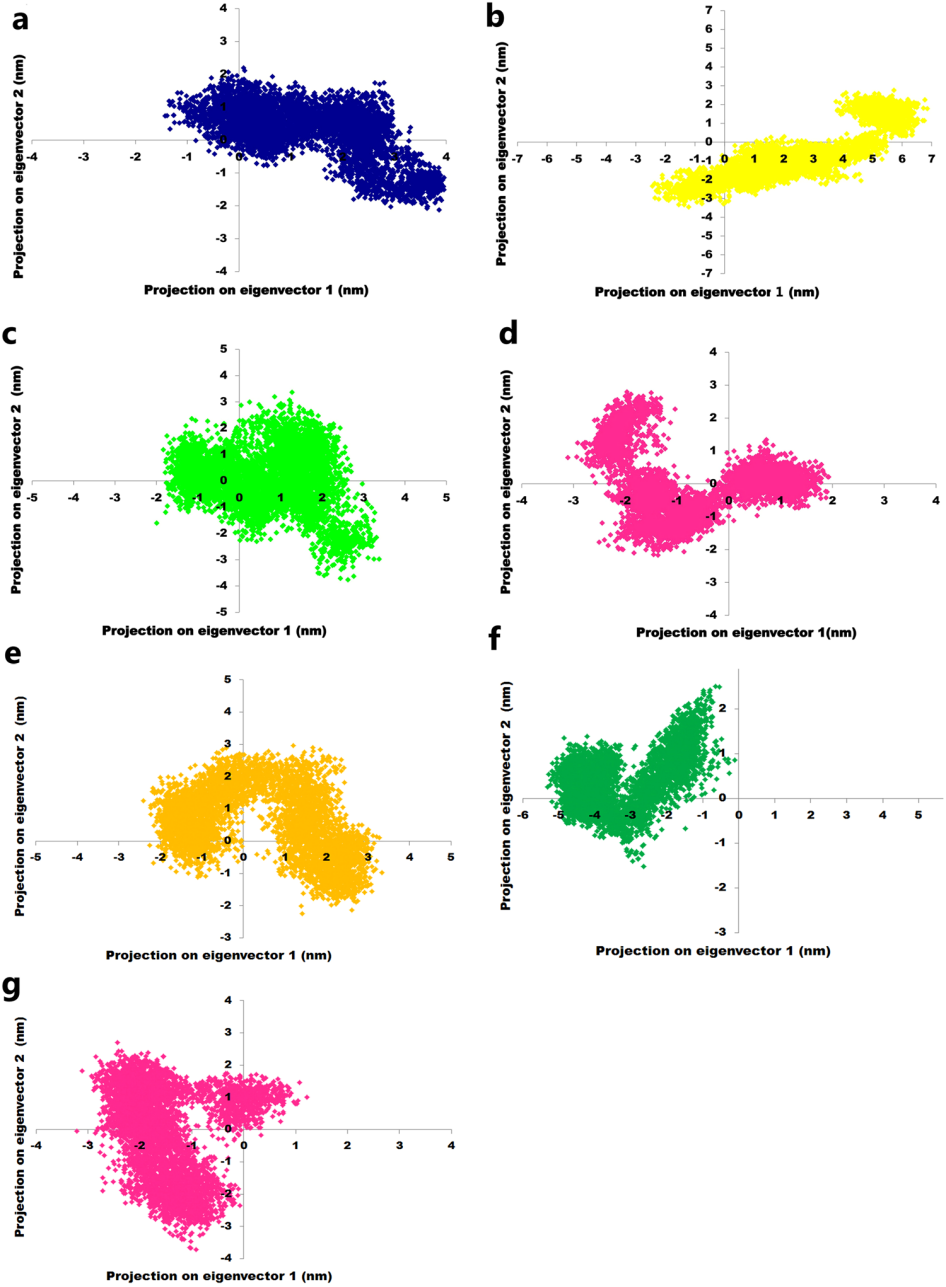
Principle component analysis plotted for the first two principle components PC1 and PC2 against time for ThyA bound with substrate dUMP and co-factor MTHF (**a**) wild-type, (**b**) R127L, (**c**) L143P, (**d**) C146R, (**e**) L172P, (**f**) A182P and (**g**) V261G mutants.

**Figure 7:**
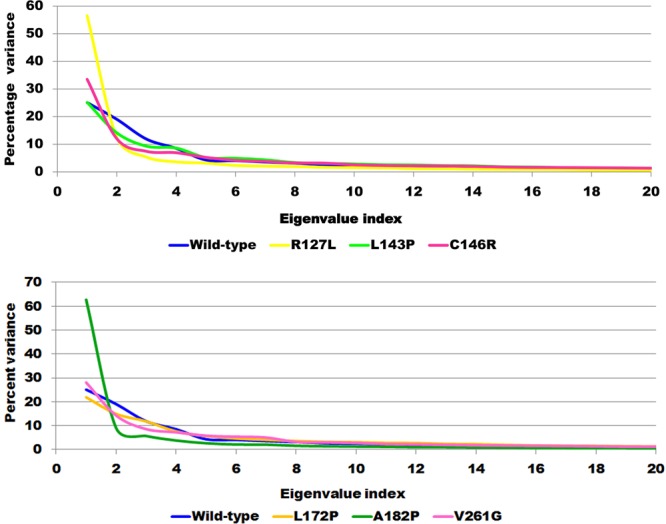
The percentage variance plot against eigenvalue index for ThyA bound with substrate dUMP and cofactor MTHF (**a**) wild-type, (**b**) R127L, (**c**) L143P, (**d**) C146R, (**e**) L172P, (**f**) A182P and (**g**) V261G mutants.

**Figure 8:**
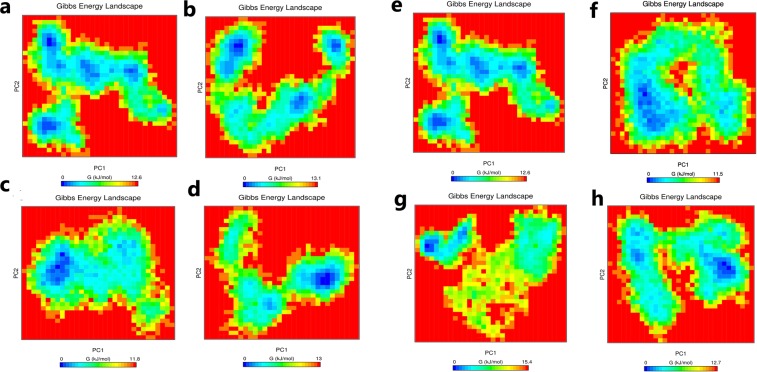
Gibbs free energy landscape calculated from first two principle component analysis for ThyA bound with substrate dUMP and cofactor MTHF (**a** and **e**) wild-type, (**b**) R127L, (**c**) L143P, (**d**) C146R, (**f**) L172P, (**g**) A182P and (**h**) V261G mutants.

The understanding of the correlation of atomic movement provides insight into enzyme and substrate interaction, particularly that arising from collective motion of the atoms, which are controlled by the secondary structure element of the proteins^24^. Covariance values can be positive or negative, which provides information about the cooperativity of motion. All diagonal elements of the symmetric 3N × 3N covariance matrix were summed and termed as trace value, which provides information about the measure of total variance (Fig. 5). The trace values calculated for the wild-type, R127L, L143P, C146R, L172P, A182P, and V261G were found to be 15.48 nm^2^, 22.51 nm^2^, 12.05 nm^2^, 9.54 nm^2^, 12.61 nm^2^, 27.66 nm^2^, and 14.02 nm^2^. Higher trace values of R127L and A182P relative to the wild-type suggested an association with the enhanced flexible behavior of the enzymes upon binding of the substrate and cofactor. It was reported that principal components (PC) reveal the most dominant internal modes of motion of a corresponding system^25^. PC analysis was applied to the backbone atoms of the wild-type and mutant ThyA enzymes bound with cofactor and substrate. PC1 must be denoted as the most important as it accounts for maximum variability in terms of internal motion of proteins in the enzyme conformation during binding of substrate and cofactor, while PC2 accounts for the remaining variability. As shown in Fig. 6, it was clearly observed that R127L and A182P mutants of ThyA occupy larger subspaces corresponding to their higher trace values. Percentages of variance against eigenvalues of the covariance matrix resulting from the simulations are shown in Fig. 7. Gibbs free energy landscape (FEL) plot was generated using PC1 and PC2 coordinates in which blue, green, and cyan represented metastable conformation with a low-energy state, while red signified high-energy protein conformation. The ∆G value wild-type, R127L, L143P, C146R, L172P, A182P, and V261G ranged from 11.5 to 15.4 kJ/mol (Fig. 8).

## Discussion

The increased drug resistance in the *Mycobacterium* is considered to be a major problem faced in the treatment of TB^26^. The folate pathway is reported to be very important for the growth and survival of the bacteria. In *M. tuberculosis*, functional ThyA catalyzes the reductive methylation of 2′-deoxyuridine-5′-monophosphate (dUMP) to thymidine-5′-monophosphate (TMP) while utilizing N5, N10-methylene-5, 6, 7, 8-tetrahydrofolate (MTHF) in folate metabolism^16^. To understand the PAS resistance mechanism, five genes involved in folate pathways (*thyA*, *dfrA*, *folC*, *folP1*, and *folP2*) and an alternative *thymine biosynthesis* gene (*thyX*) were studied in PAS-resistant Mtb clinical isolates and spontaneous mutants, respectively^14^. Among several folate pathway genes in Mtb, *thyA* was the first gene reported to be associated with PAS resistance. Previous studies have identified mutation in *thyA* gene in one-third of the PAS resistance clinical isolates and laboratory mutants^14,15^. Moreover, clinical isolates from tuberculosis patients from northern China from 2006 to 2012 showed that, among 208 isolates, 54 (26.0%) showed 24 mutations in the *thyA* gene^15^.

The identified mutations were correlated with antibiotic potency in terms of the MICs value, and a template-based (X-ray structure of the *E. coli* ThyA) three-dimensional structure of *M. tuberculosis* ThyA was constructed^14^. Further, mapping of the 24 identified point mutations in the thyA gene onto an *M. tuberculosis* ThyA homology-derived homodimer structure revealed that these mutations were in the dUMP substrate binding site, catalytic site, and folate cofactor binding site^14^. These point mutations also led to thermodynamic instability of the ThyA enzyme, confirmed with positive folding free energy value and also predicted to produce dysfunctional and unfolded polypeptide^14^.

Several studies have been conducted to investigate the mechanism of drug resistance in *M. tuberculosis* using structural dynamics and bioinformatics studies. Kumar and Sobhia (2015) demonstrated that *inhA* mutants reduced the interaction between NADH and binding site residues, causing isoniazid resistance in Mtb^27^. Similarly, various studies reported that mutation in *rpoB* gene leads to rifampicin resistance in Mtb^28–30^. Likewise, the mechanism of pyrazinamide resistance in the *pncA* gene mutants has been studied using a computational approach^31^.

Clear elucidation of the PAS resistance mechanism and ThyA conformational change is yet to be addressed specifically. Availability of the crystal structure of the Mtb ThyA enzyme provided us the opportunity to gain insight into the mechanism of emergence of drug resistance against PAS. Molecular dynamics simulations were carried out for the selected mutations with MIC values >128 µg/ml for 50 ns time period. The binding free energy of the wild-type ThyA enzyme bound with substrate and cofactor was lower (more negative) than in the mutant complexes. Accordingly, the wild-type complex representative structure clearly indicates that the Asp167, Asp166, Tyr94, His207 and Asn177 (from chain A) and Arg126 and Arg127 (from chain C) residues from the ThyA enzyme were directly involved in dUMP binding and the Ile79 and Lys49 residues were directly involved in MTHF binding. Furthermore, substantial conformational changes were observed in the L143P and V261G mutants, which would affect substrate binding affinity, which is well supported by RMSD and RMSF analysis and also Fivian-Hughes *et al*.^16^ reported that V261G mutation leads to non-functional ThyA enzyme in Mtb^16^. It is, however, worth mentioning that mutations significantly influenced the dynamic behavior of complexes, which was reflected by major disruption in the interaction network and altered motions in the mutants. Simulation results explained the reason for the strong link between PAS resistance and *thyA* gene mutations, such as wild-type, R127L, L143P, C146R, L172P, A182P, and V261G. Altogether, the results reported in the study will provide greater understanding of ThyA mutation-associated PAS resistance and will pave a path for facilitating therapeutic strategies against tuberculosis.

## Materials and Methods

### Protein structure preparation and mutant generation

ThyA protein model complex with substrate and cofactor (ThyA-dUMP-MTHF) was generated by superposition of chains A and C from 3QJ7 on the crystal structure of Mtb ThyA (PDB ID: 4FOG) bound with 5-Fluoro-dUMP and 5-Methyl-5,6,7,8-Tetrahydrofolic acid (which corresponds exactly to 5,10-methylenetetrahydrofolate)^15^. In the optimized PDB file, point mutation was inserted at selected positions using Schrödinger’s Protein Preparation Wizard^32^.

### Atomistic simulation of wild-type and mutants

MD simulations of the wild-type and six mutants were carried out using Desmond software from Schrödinger^33^. The systems were first processed and optimized using Schrödinger’s Protein Preparation Wizard^32^. The optimized systems were solvated in a cubic box with dimensions of 15Å × 15Å × 15Å using an SPC water model. Charges on the system were neutralized by adding corresponding counter ions such as Cl^−^/Na^+^ for the accurate prediction of the physical properties of a realistic system. Furthermore, all systems were relaxed and energy minimized under an NPT ensemble at 300 degrees Kelvin and pressure of 1 bar using a default protocol. Finally, well equilibrated systems were subjected to a production run of 50 nanoseconds (ns) and internal energy was recorded for every 1.2 ns. The final MD simulation run was completed in eight stages. Energy minimization using the steepest descent method was carried out in two stages, while the equilibration phase occurred in four stages before the final (step 8) production time.

Stage 1 – Task.

Stage 2 - Simulate, position Langevin dynamics NVT, T = 10 K, small timesteps, and restraints on solute heavy atoms, 100 ps.

Stage 3 - Simulate, NVT, T = 10 K, small timesteps, and restraints on solute heavy atoms, 12 ps.

Stage 4 - Simulate, NPT, T = 10 K, and restraints on solute heavy atoms, 12 ps.

Stage 5 - Solvate system.

Stage 6 - Simulate, NPT and restraints on solute heavy atoms, 12 ps.

Stage 7 - Simulate, NPT and no restraints, 24 ps.

Stage 8 - Run production MD.

### Desmond trajectory analysis

The trajectory analysis was carried out using inbuilt python script in the Desmond software package. More intense analysis was carried out by converting Desmond trajectories into Gromacs trajectories using Visual Molecular Dynamics (VMD) software^34^. Furthermore, the representative structure for all the systems was also used to perform secondary structure analysis using the VMD package as described in our previous study^35^.

### Binding pocket and residue network analysis

A cavity with specific volume and area usually located on the outer surface or interior of the protein has suitable properties for ligand binding and is referred to as a binding pocket^36^. Study of the protein binding pocket dynamics reveals significant information about the interaction specificity. To measure the influence of point mutation on the protein binding pocket, comparative binding pocket analysis for all protein systems was carried out on the final representative structure using a CASTp server at intervals of 10ns^37^.

Furthermore, residue network analysis for all seven systems was carried out using a RING 2.0 server^38^. The RING server generated networks using complex empirical re-parameterization of distance thresholds performed on the PDB, in which nodes are residue and arcs are inter- and intra-chain interactions present in the protein structure. The output xml file of the RING server was visualized using the RINalyzer and structureViz packages in Cytoscape^39^.

### Electrostatic properties calculations

The electrostatic potential differences in the wild-type and mutants were assessed through a Bluues server^40^ using a solvent probe radius, salt radius, and minimum atomic radius of 1.4 Å, 2.0 Å, and 1.0 Å, respectively at 0.15 M ionic strength and a temperature of 298.15 K. Moreover, inner and outer dielectric constants were set at 4.0 and 78.5, respectively.

### MM/GBSA binding free energy

The difference in free energy of the binding of a ligand to an enzyme and a mutant enzyme was calculated using MM/GBSA^41^. In MM-GBSA, free energy is treated as the sum of conformational energy term (i.e., MM) and solvation free energy. The MM, which, as mentioned earlier, stands for molecular mechanics, refers to the type of energy function used to calculate the potential energy of a molecular structure. These functions, usually called force fields, are classical potentials including terms describing covalent bonding, van der Waals interactions, etc. The other part, the solvation term, can be further expressed as the sum of a polar component and a non-polar contribution^42^. The latter is usually assumed to depend linearly on the solvent-accessible surface area. The binding energies between ThyA and dUMP and ThyA and MTHF in the pre- and post-MD simulated complex were computed using the MM-GBSA approach via Schrödinger Prime software^43^.

### Analyzing atomic motion and fluctuation on MD trajectories

MD simulations generate large amount of data in terms of MD trajectories. PCA is a statistical approach for extracting and analyzing the functional motion of a protein^44^. Initially, a covariance matrix of atomic fluctuation was generated using gmx covar module of gromacs. Diagonalization of the matrix yielded eigenvector and corresponding eigenvalues that describe correlation properties of the collective motion in protein from atomic fluctuation. The eigenvector corresponding to the eigenvalues is called PCA and gives direction and relative amplitude for each atomic displacement in trajectories^45^. The trajectory of an individual eigenvector displays the projections as a function of time using the gmx anaeig command. Gromacs gmx sham package were used to identify significant conformational changes in the wild-type and mutant enzyme free energy landscape.

## Supplementary information

Table S1, Table S2, Table S3, Figure S1
