# Supplementary material for: Analysis of mutations leading to para-aminosalicylic acid resistance in Mycobacterium tuberculosis
Source: Sci Rep. 2019 Sep 20;9:13617. doi: 10.1038/s41598-019-48940-5 (PMC6754364; doi:10.1038/s41598-019-48940-5)
Supplement: Supplementary file 1 — Table S1, Table S2, Table S3, Figure S1 [file 41598_2019_48940_MOESM1_ESM.docx]

**Analysis of mutations leading to *para*-aminosalicylic acid resistance in *Mycobacterium tuberculosis***

Bharati Pandey^1^, Sonam Grover^2^, Jagdeep Kaur^1^ and Abhinav Grover^3^*

^1^Department of Biotechnology, Panjab University, Chandigarh, India - 160014

^2^JH-Institute of Molecular Medicine, Jamia Hamdard, Hamdard Nagar, New Delhi 110062

^3^School of Biotechnology, Jawaharlal Nehru University, New Delhi, India - 110067

*Corresponding author

**Abhinav Grover**,

School of Biotechnology, Jawaharlal Nehru University, New Delhi, India – 110067

Tel: +91-8130738032; Fax: +91-11-26742040;

Email: [abhinavgr@gmail.com](mailto:abhinavgr@gmail.com), [agrover@jnu.ac.in](mailto:agrover@jnu.ac.in)

NUMBER OF PAGES: 4

NUMBER OF TABLES: 3

NUMBER OF FIGURES: 1

**Supplementary Tables**

**Table S1:** RMSF value for each residue for the ThyA wild-type and mutants

| **Position** | **Wild-type** | | **R127L** | | **L143P** | | **C146R** | | **L172P** | | **A182P** | | **V261G** | |
| --- | --- | --- | --- | --- | --- | --- | --- | --- | --- | --- | --- | --- | --- | --- |
|  | **Ch_A** | **Ch_C** | **Ch_A** | **Ch_C** | **Ch_A** | **Ch_C** | **Ch_A** | **Ch_ C** | **Ch_A** | **Ch_C** | **Ch_A** | **Ch_ C** | **Ch_A** | **Ch_C** |
| **127** | 0.66 | 0.68 | 0.55 | 0.76 | 0.61 | 0.83 | 0.45 | 0.53 | 0.80 | 0.63 | 0.50 | 0.60 | 0.47 | 0.60 |
| **143** | 0.87 | 1.15 | 0.80 | 0.88 | 0.69 | 0.65 | 0.62 | 0.59 | 0.90 | 0.79 | 0.64 | 0.82 | 0.80 | 0.77 |
| **146** | 0.55 | 0.75 | 0.61 | 0.88 | 0.49 | 1.16 | 0.48 | 0.70 | 0.61 | 0.74 | 0.48 | 0.86 | 0.53 | 0.72 |
| **172** | 0.66 | 0.67 | 0.67 | 0.70 | 0.48 | 0.55 | 0.50 | 0.49 | 0.53 | 0.57 | 0.49 | 0.57 | 0.59 | 0.53 |
| **182** | 0.54 | 0.43 | 0.52 | 0.64 | 0.38 | 0.51 | 0.42 | 0.43 | 0.45 | 0.56 | 0.50 | 0.84 | 0.48 | 0.51 |
| **261** | - | 2.82 | - | 1.70 | - | 1.39 | - | 1.20 | - | 1.85 | - | 9.68 | - | 1.48 |

Chain A: Ch_A and Ch_C: Chain C

**Table S2:**  Comparative secondary structure analysis of the key-residue in the ThyA wild-type and mutants

| **Position** | **Wild-type** | | **R127L** | | **L143P** | | **C146R** | | **L172P** | | **A182P** | | **V261G** | |
| --- | --- | --- | --- | --- | --- | --- | --- | --- | --- | --- | --- | --- | --- | --- |
|  | **Ch_A** | **Ch_C** | **Ch_A** | **Ch_C** | **Ch_A** | **Ch_C** | **Ch_A** | **Ch_C** | **Ch_A** | **Ch_C** | **Ch_A** | **Ch_C** | **Ch_A** | **Ch_C** |
| **127** | Helix | 3_10_ helix | Turn | Turn | Turn | Turn | Turn | Turn | Turn | Turn | Turn | Coil | Turn | Turn |
| **143** | Helix | Helix | Turn | Turn | Turn | Turn | Turn | Turn | Turn | Turn | Turn | Turn | Turn | Coil |
| **146** | Helix | Helix | Turn | Turn | Turn | Turn | Turn | Turn | Turn | Coil | Turn | Coil | Turn | Coil |
| **172** | Helix | Helix | Turn | Turn | Turn | Turn | Turn | Turn | Turn | Helix | Turn | Helix | Turn | Turn |
| **182** | Helix | Helix | Helix | Helix | Helix | Helix | Helix | Helix | Helix | Helix | Helix | Helix | Helix | Helix |
| **261** | - | Turn | - | Coil | - | Coil | - | Coil | - | Coil | - | Coil | - | Coil |

**Table S3**: Residue interaction network showing interaction of ThyA residues with dUMP and MTHF in (a) wild-type, (b) R127L, (c) L143P, (d) C146R, (e) L172P, (f) A182P and (g) V261G mutants

| **Systems** | **dUMP** |  | **MTHF** |
| --- | --- | --- | --- |
|  | **Chain A** | **Chain C** | **Chain A** |
| **Wild-type** | Trp80(2.8Å), Trp83 (3.9 Å), Tyr94 (1.9 Å), Leu143(2.0 Å), Cys146(3.7 Å), His147(3.3 Å), Gln165(3.0 Å), Arg166(1.5 Å), Ser167(1.9Å), Ala168(2.7Å), Gly173(2.7 Å), Asn177(2.3 Å), His207(2.1 Å), Tyr209(2.8 Å), Arg126(1.4 Å), | Arg126(1.6Å), Arg127(1.6 Å) | Lys48(3.8Å), Val50(2.6Å), His51(3.9Å), Ser54(1.9Å), Thr78(3.9Å), Ile79(2.2Å), Trp80(2.9Å), Trp83(2.4Å), Leu143(3.0Å), Asp169(2.5Å), Phe171(3.0Å), Leu172(2.0Å), Gly173(2.5Å), Pro175(3.9Å), Phe176(2.1Å), Tyr209(3.5Å), Pro224(3.9 Å), Lys258(3.8 Å), Ala259(2.3 Å) |
| **R127L** | Trp80(2.3Å), Trp83(2.7Å), Cys146(3.9Å), His147(2.9 Å), Gln165(3.6Å), Arg166(1.6 Å),Ala168(2.5Å),Asp169(2.6Å)Gly173(3.8Å),Asn177(3.8Å),His207(2.1Å),Tyr209(2.2Å), Arg126(1.7 Å), Leu127(4.0 Å) | Arg126(1.7Å), Leu127(4.0Å) | Lys49(1.7Å), Val50(3.9Å), His51(1.9Å), Ser54(3.2Å), Val55(3.9 Å), Phe176(2.4 Å), Ala256(2.8Å) |
| **L143P** | Thr26(3.9Å), Trp80(2.6Å), Pro144(3.2Å), Cys146(3.4Å), His147(2.9Å), Gln165(2.8 Å), Arg166(1.5 Å), Ala168(2.4 Å), Asp169(1.7Å), Val174(2.6Å), Asn177(3.7Å), His207(2.0Å), Tyr209(1.8Å), Arg126(1.7Å), Leu127(4.0Å) | Arg126(3.5Å) | Lys48(2.3 Å), Trp80(2.6 Å), Glu82(2.5Å), Trp83(2.1Å), Phe171(2.9Å), Leu172(2.4Å), Gly173(2.1Å), Phe176(2.1Å), Tyr209(3.0Å), Ala256(2.3Å), Ile257(2.8Å), Ala259(3.4Å) |
| **C146R** | Pro144(3.4 Å), Cys146(3.4Å), His147(3.7Å), Gln165(3.4 Å), Arg166(1.4 Å), Ser167(3.5 Å), Ala168(3.0 Å), Asp169(1.9 Å), Leu172(3.9), Gly173(2.8 Å), Val174(3.1 Å), Asn177(2.2 Å), His207(2.4 Å), Tyr209(2.2 Å), Pro224(3.9 Å), | Arg126(2.2Å) | Lys48(2.4 Å), Lys49(3.7Å), Val50(2.6Å), His51(2.0Å), Ser54(3.4Å), Glu58(3.5Å), Ile79(2.4Å), Trp80(2.6Å), Trp83(2.2 Å), Arg146(3.6Å), Phe171(2.9Å), Leu172(2.5Å), Gly173(2.8Å), Phe176(3.8Å), Asn177(3.4Å) |
| **L172P** | Ile79(2.7Å), Trp80(3.6Å), Trp83 (3.3Å), Cys146(1.5Å), His147(3.4Å), Arg166(1.6Å), Ala168(3.1 Å), Gly173(2.5Å), Asn177(3.9Å), His207(2.0Å), Tyr209(1.8Å), Arg126(1.7Å), Leu127(4.0Å), | Arg126(3.5Å) | Lys48(3.0 Å), Lys49(1.8Å), Val50(3.3Å), His51(3.5Å), Ser54(3.0Å), Thr78(2.3Å), Glu82(2.2 Å), Trp83(3.8Å), Pro172(2.7Å), Gly173(3.9 Å), Phe176(3.9Å), Ala256(3.5Å) |
| **A182P** | Ile79(2.9 Å), Trp80(3.5 Å), Trp83 (2.3 Å), Pro144(3.8 Å), Cys146(1.9Å), Gln165(3.3Å), Arg166(1.6Å), Ser167(2.4Å), Ala168(2.6Å), Asp169(2.1Å), Leu172(3.9Å), Gly173(2.5Å), Phe176(3.4 Å), Asn177(3.7Å), His207(3.0Å), Tyr209(4.0Å), Arg126(1.7Å), Leu127(4.0Å), | Arg126(3.5Å) | Lys48(2.1Å), Lys49(1.8Å), Val50(3.2Å), His51(3.7Å), Ser54(2.7Å), Thr78(2.4Å), Trp83(3.7Å), Pro172(2.7Å), Gly173(3.1Å), Phe176(3.1Å), Ala256(3.5Å) |
| **V261G** | Glu58(3.8Å), Phe62(3.9Å), Trp80(3.5Å), Trp83 (1.6Å), Tyr94(3.4Å), Cys146(2.5Å), His147(2.9Å), Gln165(2.9Å), Arg166(1.6Å), Ser167(3.4Å), Ala168(2.9Å), Asp169(2.3Å), Gly173(2.7Å), Asn177(3.7Å), His207(3.0Å), Tyr209(4.0Å), Arg126(1.7Å), Leu127(4.0Å), | Arg126(3.5Å) | Lys48(2.6Å), Lys49(2.0Å), Val50(3.4Å), His51(2.6Å), Thr78(2.2Å), Ile79(2.3Å), Glu82(2.5Å), Trp83(3.7Å), Leu172(2.7Å), Phe176(2.3Å), Ala256(3.5Å), Ile257(2.9Å) |

*Residues interacting distance ≤ 4Å were listed.


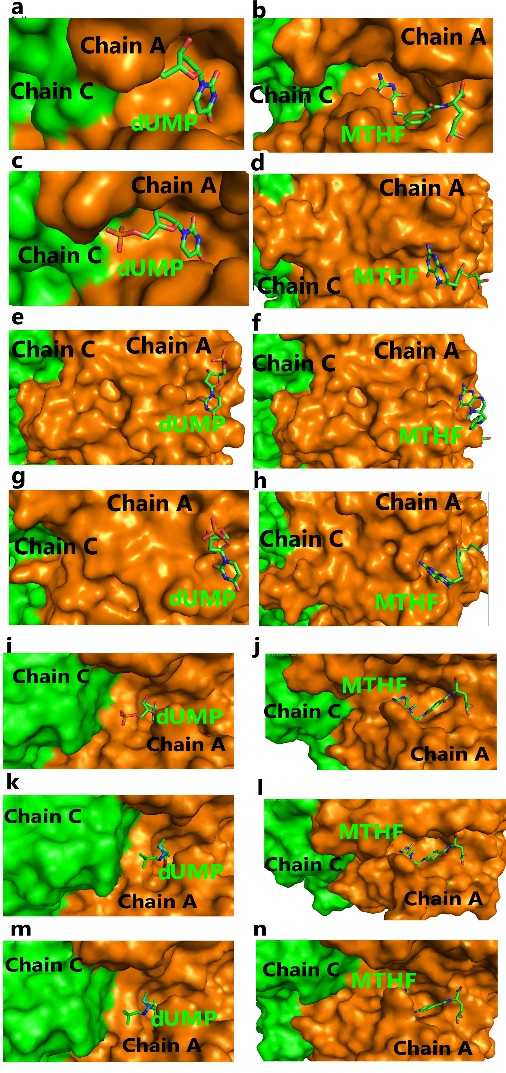


**Figure S1**: Surface representation of dUMP and MTHF with the residues of ThyA in wild-type (a and b), R127L (c and d), L143P (e and f), C146R (g and h), L172P (I and j), A182P (k and h) and V261G (m and n) mutants.
